# Melatonin reduces LH, 17 beta-estradiol and induces differential regulation of sex steroid receptors in reproductive tissues during rat ovulation

**DOI:** 10.1186/1477-7827-9-108

**Published:** 2011-08-02

**Authors:** Luiz Gustavo A Chuffa, Fábio RF Seiva, Wagner José Fávaro, Giovana R Teixeira, João PA Amorim, Leonardo O Mendes, Beatriz A Fioruci, Patrícia Fernanda F Pinheiro, Ana Angélica H Fernandes, Janete AA Franci, Flávia K Delella, Marcelo Martinez, Francisco E Martinez

**Affiliations:** 1 Department of Structural and Cellular Biology, Institute of Biology, Universidade Estadual de Campinas - UNICAMP, Campinas-SP 13083-863, Brazil; 2 Department of Anatomy, Bioscience Institute, UNESP - Univ. Estadual Paulista, Botucatu-SP 18618-000, Brazil; 3 Department of Chemistry and Biochemistry, Bioscience Institute, UNESP - Univ. Estadual Paulista, Botucatu-SP 18618-000, Brazil; 4 Department of Morphology and Pathology, UFSCar - Universidade Federal de São Carlos, São Carlos-SP 13565-905, Brazil; 5 Department of Morphology, Stomatology and Physiology, USP - Universidade de São Paulo, Ribeirão Preto-SP 14040-900, Brazil

## Abstract

**Background:**

Melatonin is associated with direct or indirect actions upon female reproductive function. However, its effects on sex hormones and steroid receptors during ovulation are not clearly defined. This study aimed to verify whether exposure to long-term melatonin is able to cause reproductive hormonal disturbances as well as their role on sex steroid receptors in the rat ovary, oviduct and uterus during ovulation.

**Methods:**

Twenty-four adult Wistar rats, 60 days old (+/- 250 g) were randomly divided into two groups. Control group (Co): received 0.9% NaCl 0.3 mL + 95% ethanol 0.04 mL as vehicle; Melatonin-treated group (MEL): received vehicle + melatonin [100 μg/100 g BW/day] both intraperitoneally during 60 days. All animals were euthanized by decapitation during the morning estrus at 4 a.m.

**Results:**

Melatonin significantly reduced the plasma levels of LH and 17 beta-estradiol, while urinary 6-sulfatoximelatonin (STM) was increased at the morning estrus. In addition, melatonin promoted differential regulation of the estrogen receptor (ER), progesterone receptor (PR), androgen receptor (AR) and melatonin receptor (MTR) along the reproductive tissues. In ovary, melatonin induced a down-regulation of ER-alpha and PRB levels. Conversely, it was observed that PRA and MT1R were up-regulated. In oviduct, AR and ER-alpha levels were down-regulated, in contrast to high expression of both PRA and PRB. Finally, the ER-beta and PRB levels were down-regulated in uterus tissue and only MT1R was up-regulated.

**Conclusions:**

We suggest that melatonin partially suppress the hypothalamus-pituitary-ovarian axis, in addition, it induces differential regulation of sex steroid receptors in the ovary, oviduct and uterus during ovulation.

## Background

Melatonin (*N*-acetyl-5-methoxytryptamine) also known as "chemical expression of darkness" is an indolamine produced by pineal gland and secreted in a circadian manner during the night [1]. It is indisputable that melatonin has been potentially implicated as a therapeutic agent in several conditions. In mammals, melatonin can affect the reproductive function through activation of receptor sites within the hypothalamic-pituitary-gonadal axis [2]. Previous evidence has suggested that changes consistent with inhibition of GnRH release occur after melatonin implants [3]. Melatonin is found inside ovarian follicles [4], thus proving its direct action in ovarian function. It has also been proposed that pre-ovulatory follicles contain high amount of melatonin which were indirectly linked to the 17 β-estradiol (E2) and progesterone (P4) synthesis [5]. In melatonin-deprived rats, an increased estrous frequency was inversely related to the luteinizing hormone (LH) and follicle-stimulating hormone (FSH) levels [6]. According to Soares et al. [7], the low melatonin levels lead to a reduction of P4 and its receptors while increasing E2 levels. Moreover, it was reported that melatonin might decrease E2 levels during the premenopausal period [8]. Most studies investigating the mechanism(s) by which melatonin modulates the reproduction have focused mainly in the pituitary and hypothalamus or in evaluating the effects of pinealectomy, with little attention devoted to the relationship between exogenous melatonin treatment and female reproductive tissues during ovulation. Furthermore, these reproductive actions promoted by long-term melatonin administration in a non-seasonal breeder (e.g. rat) are yet poorly understood.

More recently, it was noted that administration of melatonin at night induces prolonged diestrous phase in normal rats [9,10]. There seem to be little doubt that exogenous melatonin restores the basal gonadotropin concentrations (FSH and LH) in aged rats as similar to young rats [11], also having a stimulatory effect on E2 levels and pituitary responsiveness to LHRH [12]. Nevertheless, the effects of melatonin on reproductively active rats, at the timing of ovulation, remain a matter of debate.

In reproductive system, melatonin may interact with sex steroids [13-15]. It is well-known that sex steroid receptors might regulate a variety of physiological responses in the ovary, oviduct and uterus tissue when they are activated [13,16,17]. Estrogen receptor (ER), a member of the nuclear receptor superfamily, has two functional isoforms designated as ER-α and ER-β [18]. In ovaries, the granulosa cells express higher levels of ER-β than ER-α, while ER-β is reportedly expressed at lower levels in uterus [19]. Importantly, a repetitive loss of ER-β expression or a decrease in ER-β/ER-α ratio is linked to ovarian epithelial tumorigenesis [20]. Other study showed a decreased number of uterine estrogen receptor with concomitant increase of PR after 15-day melatonin treatment [13]. However, none have evaluated the role of melatonin considering different steroid receptors isoforms. Despite of considerable effort, the effects of long-term melatonin focused on reproductive hormones and its specific receptors involving the ovaries, oviducts and uterus are not well discussed.

Progesterone receptors (PRs), one of the well-characterized estrogen-regulated genes, are expressed as PR-A and PR-B isoforms [21]. PRA has a transactivation role in some cells whereas it functions as a repressor of PRB (heterodimer form) and androgen receptor [16]. Although it has long been emphasized that E2 up-regulates PR, little is known as to whether PRA and B expression is modulated by either E2 or melatonin. Furthermore, E2 seems to alter expression from PRB to PRA dominancy in oviduct and uterus [22,23]. Not surprisingly, PRA, but not PRB expression, is necessary and sufficient for ovulation process [17]. More recently, melatonin significantly increased P4 as well as the number of total PR in ovarian tissue at proestrus [15]. Otherwise, Soares Jr. et al. [7] found a diminution of 6-sulfatoximelatonin (STM) metabolite and PR levels after pinealectomy surgery. To date, the melatonin effects on selective PRA and PRB have not been demonstrated.

Melatonin signals through at least two G protein-coupled receptors, the MTR1 and MTR2 membrane receptors, or via putative cytoplasmatic/nuclear sites mediating the physiological responses [24,25]. Among other actions, MTR1-binding melatonin is thought to cause down-regulation of both ER-α protein and ER-α mRNA [26] and, alternatively, it may inhibit the ligation of E2-ER complex to the estrogen response elements (ERE) on DNA [14,26], thus dampening the E2-mediated effects. Since melatonin is a potential agent controlling the reproduction, its long-term effects related to reproductive tissues, at estrous phase, have never been identified through MT1R receptors.

Therefore, the present study was undertaken to verify whether exposure to long-term melatonin is able to cause reproductive hormonal disturbances as well as their role upon sex steroid receptors in the rat ovary, oviduct and uterus during ovulation process.

## Methods

### Animals and experimental design

Twenty-four adult female rats (*Rattus norvegicus albinus*), 60 days old (± 250 g) were obtained from the Department of Anatomy, Bioscience Institute, UNESP - Univ Estadual Paulista, Campus of Botucatu. All animals were housed in polypropylene cages (43 cm × 30 cm × 15 cm) with laboratory-grade pine shavings as bedding and also maintained under controlled room temperature (23 ± 1°C) and lighting conditions (12 L, 12 D photoperiod, lights switched on at 6 a.m). Initially, the animals were randomly divided into two experimental groups (n = 12/group). Control group: rats fed standard chow and tap water *ad libitum *and receiving 95% ethanol 0.04 mL + 0.9% NaCl 0.3 mL (1:7 v/v) as vehicle; Melatonin-treated group: rats fed standard chow and tap water *ad libitum *receiving vehicle + melatonin. At 90 days old, females started to receive successive doses of melatonin over 60 consecutive days. After melatonin treatment period, all rats were monitored by vaginal swabs in a dark room using a red dim illumination, and during the early morning of estrus (timing of ovulation) at 4 a.m (or Zeitgeber Time, (ZT) 22, corresponding to the environmental circadian time) they were anesthetized and euthanized by decapitation for further analysis. Experimental protocols were previously accepted by Ethical Committee of the Institute of Bioscience/UNESP, Campus of Botucatu, SP, Brazil (Protocol n° 85/07).

### Procedures of melatonin administration

Successive doses of melatonin [100 μg/100 g BW] (M-5250, purchased from Sigma Chemical, St Louis, MO) were dissolved in 95% ethanol 0.04 mL, using 0.9% NaCl solution as a vehicle [9]. The intraperitoneal infusions (only vehicle or vehicle + melatonin) were daily administered between 18:30 - 19:00 p.m (ZT 13).

### Urine and reproductive organs collection

In the evening before they were killed, all animals received the last injection of melatonin and they were kept inside metabolic cages (Techniplast, Exton, PA, USA) by 10 h in order to collect individual urine samples. Thereafter, all samples were centrifuged at 10,000 × g for 20 min at 4°C and stored at - 20°C. On the next day and after sacrifice, all reproductive organs (ovaries, oviducts and uterine horns) were entirely dissected and weighed for further assays.

### Sex hormones assay

Blood samples were collected from the trunk of decapitated rats into heparinized tubes. Afterwards, plasma was obtained by centrifugation at 1,200 × g for 15 min at 4°C and stored at - 20°C until assayed by radioimmunoassay (RIA). Plasma samples were assayed for FSH and LH by double-antibody RIA with specific kits provided by the "National Institute of Arthritis, Diabetes, Digestive and Kidney Diseases" (NIADDK, Baltimore, MD, USA). The FSH primary antibody was anti-rat FSH-S11, and the standard FSH-RP2. The antiserum for LH was LH-S10 using RP3 as reference. The lower limit of detection for FSH and LH was 0.2 ng/mL and the intra-assay coefficient of variation was 3% and 4%, respectively. Plasma concentrations of E2 and P4 were determined using Estradiol and Progesterone Maia kits (Biochem Immunosystems, Serotec, Italy). The lower detection limit and the intra-assay coefficient of variation were respectively 7.5 pg/ml and 2.5% for E2 and 4.1 ng/ml and 3.7% for P4. All samples were measured in duplicate and at different dilutions, if necessary. In order to prevent interassay variation, all samples were assayed in the same RIA.

### Determination of plasma melatonin and urinary 6-sulfatoximelatonin (STM)

Melatonin was initially extracted from plasma (n = 12 samples/group) using methanol HPLC grade followed by separation into columns Sep-Pak Vac C-18, reverse phase, 12.5 nm (Water Corporation, Milford, Massachusetts, USA). Thereafter, 50 μL of reconstituted samples were assayed with coat-a-count melatonin ELISA kits and measured photometrically at a wavelength of 405 nm. The intra-assay coefficient of variation was 3%. Urinary 6-STM (a metabolite of melatonin) was assayed with solid-phase melatonin sulfate ELISA kits and, finally, read at 450 nm. The intra-assay coefficient of variation was 5.2%. Samples were double assayed at the time to avoid interassay variations. All reagents and microtiter plate were provided by IBL (IBL International, Hamburg, Germany).

### Western blotting analysis and protein quantification

After 60 days of melatonin treatment (100 μg/100 g BW/day), the ovaries, oviducts and uterine horns were rapidly removed and tissue samples of 50 mg were immediately frozen in liquid nitrogen and stored at -80°C. All tissues were homogenized with RIPA lysis buffer (Pierce Biotechnology, Rockford, IL, USA), 10X (0.5 M Tris-HCl, 1.5 M NaCl, 2.5% deoxycholic acid, 10% NP-40, 10 mM EDTA, pH 7.4) and protease inhibitor cocktail (Sigma Chemical Co.) using a homogenizer (IKA^® ^T10 basic Ultra, Staufen, Germany). Aliquots containing 1:10 (v/v) of Triton X-100 were added to homogenates and samples were placed on dry ice under agitation by 2 h in order to improving extraction. These suspensions were centrifuged at 21,912 × g for 20 min at 4°C and the pellet discarded. The protein concentrations were measured by the Bradford micro-method for colorimetric determination. Total proteins were dissolved in 1.5 × sample buffer previously described by Laemmli and used for SDS-PAGE (Bio-Rad Laboratories, Hercules, CA, USA). Equal amounts of protein (70 μg) of each sample were loaded per well onto preformed gradient gels, 4-12% acrylamide (Amersham Biosciences, Uppsala, Sweden) with a Tris-glycine running buffer system for electrophoresis (60 mA fixed during 2 h). After electrophoresis, total proteins were electro-transferred (200 mA fixed by 1 h 30 min) onto 0.2 μm nitrocellulose membranes in a Tris-glycine-methanol buffer. Kaleidoscope Prestained Standards (Bio-Rad) were used as molecular weight markers. Thereafter, the membranes were blocked with TBS-T solution containing 3% BSA at room temperature (RT) for 60 min and then incubated at 4°C overnight with rabbit primary antibody AR-N20 anti-androgen receptor (AR); rabbit clone E115 anti-ERα; rabbit clone 68-4 anti-ERβ; mouse monoclonal [C262] anti-PRA and PRB and rabbit polyclonal anti-MT1R (dilutions of 1:1000; 1:250; 1:500; 1:350; 1:500; 1:500 were carried out at 1% BSA, respectively). This was followed by washing 3 × 5 min in TBS-T solution and then incubated for 2 h at RT with rabbit or mouse HRP-conjugated secondary antibodies (diluted 1:1000 in 1% BSA; Sigma, St. Louis, MO, USA). After sequential washing with TBS-T, signals were enhanced and peroxidase activity was finally detected by mixing 10 mL PBS, 8 μl H_2_O_2 _and 0.02 g diaminobenzidine (DAB) chromogen (Sigma Chemical Co.). Immunoreactive bands of each protein (arbitrary units) were obtained from separate blots of six rats/group using image analysis software (NIS-Elements, Advanced Research, Nikon). β-actin was used as an endogenous control and all results were expressed as mean ± SEM. Immunoblotting concentrations (%) were represented as optical densitometry values (band intensity/β-actin ratio).

### Statistical analysis

Data of plasma FSH, LH, E2, P4, melatonin, urinary 6-SMT and western blotting analysis were performed by Student's t test with independent samples. Statistical significance was set at *P *< 0.05 and significant results are expressed as mean ± SEM. The statistical software used was *GraphPad Instat version 4 and Sigma Plot version 11.0 *for graphic design.

## Results

### Plasma sex hormones, melatonin and urinary 6-STM levels

**Figure 1:**
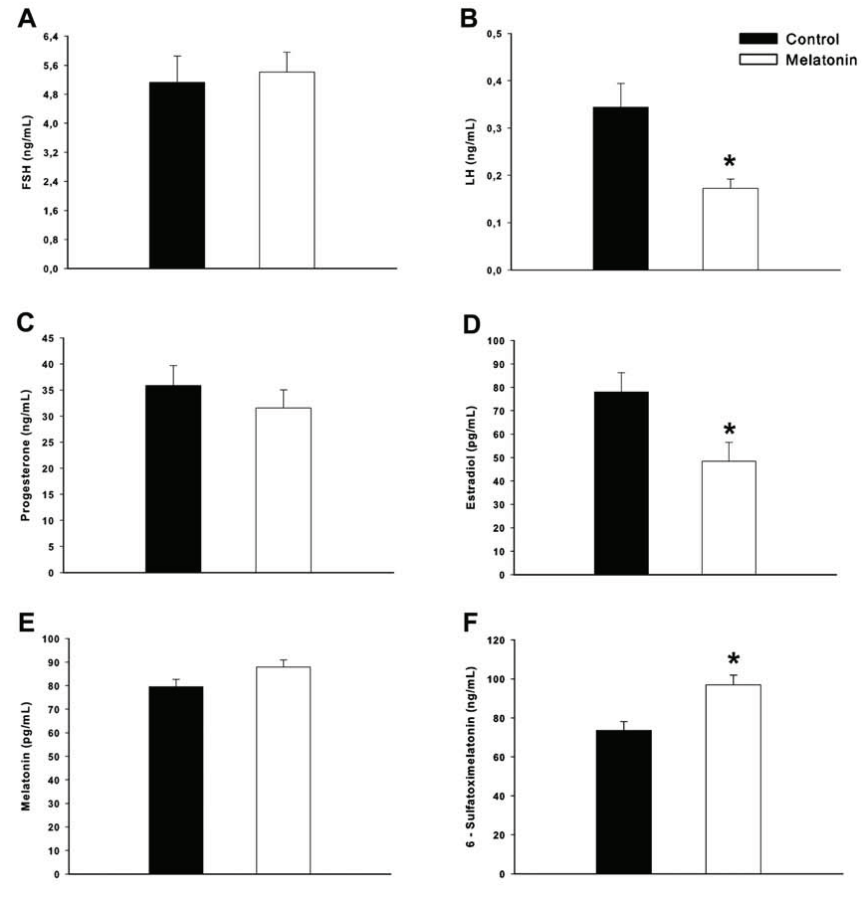
**Hormonal profile after 60-day melatonin treatment at morning estrus**. **(A) **Plasma FSH levels (ng/mL), **(B) **Plasma LH levels (ng/mL), **(C) **Plasma P4 levels (ng/mL), **(D) **Plasma E2 levels (pg/mL), **(E) **Plasma melatonin levels (pg/mL), **(F) **urinary 6-STM levels (ng/mL). Values are expressed as mean ± SEM (N= 12 animals/group). * p < 0.05 vs. control group.

After eight weeks of treatment, total LH and E2 levels were reduced in melatonin-treated rats (p < 0.05). Conversely, FSH and P4 levels had not been influenced by melatonin at the estrus phase (p > 0.05). These data confirmed our previous reports in which long-term melatonin administration leads to a reduced ovarian mass and prolonged metaestrus and diestrus duration, without blocking ovulation (recently published data). Additionally, there was no evidence for increased plasma melatonin levels in animals receiving the treatment, but the urinary 6-STM levels were significantly higher at the morning of estrus (p < 0.01; Figure 1A-F).

### Analysis of ovarian AR, ER-α, ER-β, PRA, PRB and MT1R levels after treatment

**Figure 2:**
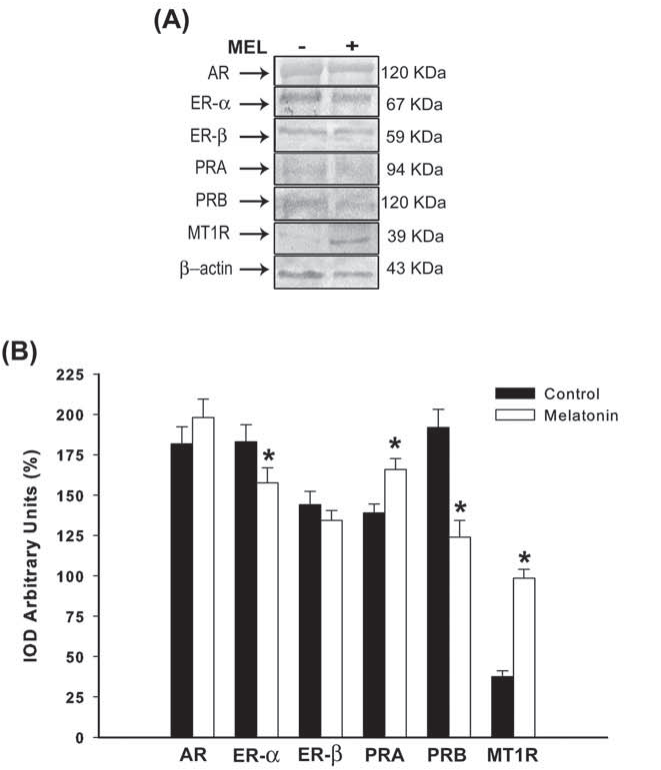
**Analysis of ovarian receptors**. **(A) **Representative Western blotting analysis of androgen receptor (AR), estrogen receptor (ER-α and ER-β), progesterone receptor (PRA and PRB) and melatonin receptor (MT1R) in ovarian tissue of rats receiving melatonin [100 μg/100 g B.W]. Indicated concentrations of each total protein (70 μg extracted from a pool of 6 organs/group) were used to detect specific protein expression levels in the blots (upper panel). **(B) **Densitometry values for AR, ER-α, ER-β, PRA, PRB and MT1R levels were studied following normalization to the house-keeping gene (β-actin). All results are expressed as mean ± SEM (N= 6 animals/group). * p < 0.05 vs. control group.

Sex steroid receptors in reproductive female tract were differentially expressed at the end of melatonin treatment. In the ovarian tissue, despite of AR and ER-β levels were not affected along the treatment, melatonin significantly reduced ER-α and PRB levels (p < 0.05; Figure 2A, B), beyond the ER-α/ER-β ratio (melatonin 1.17 ± 0.2 *vs *control 1.27 ± 0.3). Moreover, it was observed that melatonin induced significant overexpression of PRA subunit and of its own receptor MT1R (p < 0.01; Figure 2A, B). There was also an increase of the PRA/PRB ratio after melatonin treatment (melatonin 1.34 ± 0.6 *vs *control 0.73 ± 0.5).

### Analysis of oviduct AR, ER-α, ER-β, PRA, PRB and MT1R levels after treatment

**Figure 3:**
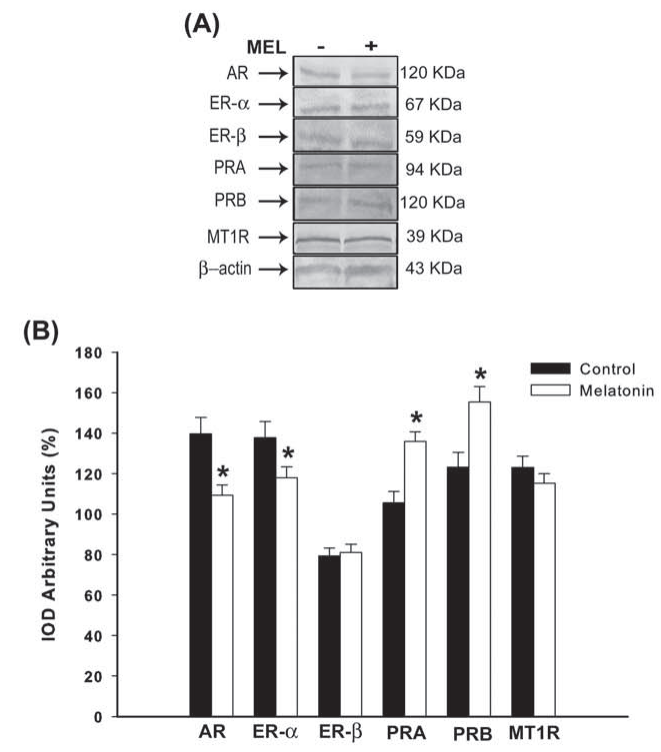
**Analysis of oviduct receptors**. **(A) **Representative Western blotting analysis of androgen receptor (AR), estrogen receptor (ER-α and ER-β), progesterone receptor (PRA and PRB) and melatonin receptor (MT1R) in oviduct tissue of rats receiving melatonin [100 μg/100 g B.W]. Indicated concentrations of each total protein (70 μg extracted from a pool of 6 organs/group) were used to detect specific protein expression levels in the blots (upper panel). **(B) **Densitometry values for AR, ER-α, ER-β, PRA, PRB and MT1R levels were studied following normalization to the house-keeping gene (β-actin). All results are expressed as mean ± SEM (N= 6 animals/group). * p < 0.05 vs. control group.

Regarding to the oviduct tissue, expressions of AR and ER-α, in addition to ER-α/ER-β ratio (melatonin 1.45 ± 0.8 *vs *control 1.73 ± 0.6) were significantly lower in melatonin-treated group (p < 0.05), while both PRA and PRB subunits had a remarkable increase after melatonin treatment (p < 0.01; Figure 3A, B). No significant PRA/PRB ratio was seen between the groups (melatonin 0.87 ± 0.2 *vs *control 0.85 ± 0.4). Furthermore, the oviduct ER-β and MT1R levels kept unchanged in the presence of melatonin (Figure 3A, B).

### Analysis of uterine AR, ER-α, ER-β, PRA, PRB and MT1R levels after treatment

**Figure 4:**
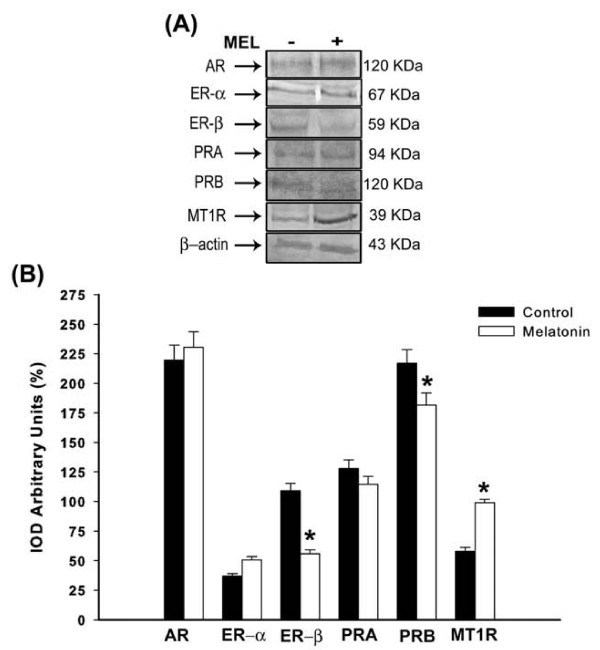
**Analysis of uterine receptors**. **(A) **Representative Western blotting analysis of androgen receptor (AR), estrogen receptor (ER-α and ER-β), progesterone receptor (PRA and PRB) and melatonin receptor (MT1R) in uterus tissue of rats receiving melatonin [100 μg/100 g B.W]. Indicated concentrations of each total protein (70 μg extracted from a pool of 6 organs/group) were used to detect specific protein expression levels in the blots (upper panel). **(B) **Densitometry values for AR, ER-α, ER-β, PRA, PRB and MT1R levels were studied following normalization to the house-keeping gene (β-actin). All results are expressed as mean ± SEM (N= 6 animals/group). * p < 0.05 vs. control group.

Following to uterus tissue, there were no differences for AR, ER-α and PRA levels (p > 0.05; Figure 4A, B). Although melatonin had significantly reduced the ER-β and PRB subunits in the uterine tissues, its selective receptor MT1R was clearly overexpressed (p < 0.05; Figure 4A, B). Moreover, in contrast to ovary and oviduct tissue, melatonin significantly increased the uterine ER-α/ER-β ratio (melatonin 0.90 ± 0.2 *vs *control 0.34 ± 0.4) but not the PRA/PRB ratio (melatonin 0.63 ± 0.6 *vs *control 0.59 ± 0.5).

## Discussion

The present study found that melatonin is able to reduce LH and E2, but not FSH and P4 levels at estrous. Although melatonin may act as a synchronizer of the reproductive function, the cellular and molecular characteristics of melatonin binding sites are so far unknown. It seems obvious that melatonin does not act directly on GnRH neurons [27,28] but, instead, exert indirect actions on *Kiss 1*/GPR54 system responsible for controlling reproduction via neural-axis by inducing low circulating gonadotropins and sex steroids levels [27,29]. In this context, the long-term melatonin treatment may be linked to the phenotype of hypogonadotropic hypogonadism, evidenced by loss of ovarian mass, as previously demonstrated by our group [9]. It has been proposed that preovulatory LH surge, until the onset of estrus, depends on the lowest melatonin levels [30,31] where increased E2 could suppress its production. It is also known that human preovulatory follicles contain amounts of melatonin in a concentration higher than those in the circulating serum, where it strongly regulates the steroid synthesis by the gonads [32]. Ultimately, melatonin may drastically influence the success of ovulation.

Indeed, exogenous melatonin induces a decrease in LH surge, blocking ovulation and luteal phase with increase in P4 levels, without affecting FSH or E2 levels [33]. Melatonin was also seen to inhibit steroidogenesis by altering cAMP levels through a direct action on theca or granulosa cells of the follicles [32,34]. This dual effect of melatonin allowed us to believe that low E2 levels can be associated with direct inhibition of pathway for E2 biosynthesis since FSH levels was unchanged. Furthermore, a positive feedback on LH secretion does not occur when E2 levels are low, thus explaining, in part, the hormonal disturbances in female reproduction caused by melatonin. Melatonin also regulates the expression and activity of aromatase [35], acting as a selective estrogen enzyme modulator, and further contributing to a decrease in E2 levels. Following the treatment, although the urinary 6-STM levels were raised at morning estrus, plasma melatonin levels were unchanged. This is due to the short half-life of melatonin, where it is rapidly converted into 6-STM prior to elimination. In accordance to Graham et al. [36], the increased 6-STM level is a good biomarker to predict the effectiveness of treatment.

The activity of melatonin directly influencing the ovary function and estrous cycle was first described by Wurtman et al. [37] and as expected, similar findings were previously confirmed by our group [9,38]. Recently, Adriaens et al. [39] demonstrated that melatonin increased P4 and androgen production in mouse preantral follicles. These contradictory results are partially due to different melatonin concentration, time and route of administration and period of estrus stage evaluation. Brzezinski et al. [40] reported that melatonin itself has no effect on basal P4 productions, but when combined with LH analogues, melatonin potentiated the stimulatory effect on intraovarian P4 production. Our data corroborate those findings, in which P4 was unaltered by melatonin treatment and even LH levels were insufficient to produce activity on P4 secretion. The present study showed that long-term melatonin is able to reduce the ER-α and PRB ovarian levels while increasing PRA and its receptor MT1R at morning estrus. Indeed, ER-α seems to be activated when intracellular cAMP is elevated after non-transcriptional mechanisms mediated by estrogens [41]. Alternatively, melatonin acting through membrane-bound G protein-coupled MT1 receptor can inhibits adenylate cyclase activity, thus decreasing cAMP levels [42]. This reduction may be a direct effect by which melatonin decreases E2-induced ER-α transcriptional activity. As a favorable condition, the reduction in ER-α/ER-β ratio represents a protective action of melatonin against estrogen-dependent tumor. Both PRA and PRB have been shown to function as ligand-dependent repressors of ER-mediated transcriptional activity [43]. Furthermore, PRA may act as a transdominant inhibitor of PRB and AR gene expression [44]. In this context, melatonin treatment might be accentuating PRA activity, thereby providing a negative regulation of ER-α and PRB expression. Since PRA isoform is essential for ovulation to occur [17], the long-term melatonin treatment could delay but not abolish the ovulation, as we had already been noted. Curiously, melatonin-deprived rats had lower expression of P4 and PR than controls [7,15], thus proving that melatonin is a key factor in PR regulation. Previous study has indicated that melatonin binding receptor is high during estrus, proestrus and diestrus, in contrast to low levels in metaestrus when E2 and P4 are reduced [45,46]. Thus, it allows us to conclude that both E2 and P4 regulate MT1 receptor binding activity.

We demonstrated for the first time that total expression of oviduct PRA and PRB was enhanced while AR and ER-α decreased after melatonin treatment. Generally, PRB is transcriptionally more active than PRA [47], and it is well documented that PRA acts as a repressor of PRB-dependent activation genes and, likewise, it inhibits the transactivation of AR [16]. Surprisingly, oviduct PRB has been up-regulated after melatonin exposure. It is likely that melatonin promoted a differential effect upon its regulatory mechanism(s) independently of either P4 or PRA functions. Moreover, the distinct transactivation properties, including presence or absence of the PRB-specific AF-3 domain, are probably due to the broad repertoire of physiological responses to P4 [48]. Nevertheless, the regulation of sex steroid receptors in oviduct is not yet fully clarified. Similarly to the ovary, melatonin led to a downregulation of oviduct ER-α through its direct effect or indirectly by the fall in E2 levels. Hence, an inverse ER-α/ER-β ratio also brings up a positive action of melatonin to the oviduct. The oviduct MT1R was not affected over the treatment, showing that, in fact, the ovary and uterus are more responsive to the effects of melatonin mediated by MT1R. Besides that, it seems plausible that melatonin-induced changes occur through different signaling pathway. It is well emphasized that melatonin may exert its physiological function by binding to melatonin receptors or even through nuclear signaling involving RZR/ROR receptors [49]. However, additional studies are needed for a better understanding of melatonin binding sites.

In this study, the uterine ER-β and PRB was down-regulated whereas MT1R was up-regulated. It is established that E2 and P4 acts on the uterus by an interdependent regulation of ER and PR [50,51]. Noticeably, it has been suggested that E2 decreases the expression of uterine ER but not PR, while P4 reduces the levels of both receptors [52]. Taking into account that E2 levels, which are responsible for increasing PR levels, were suppressed by the treatment, our results could be explained, in part, by the down-regulation of uterine PRB expression. In this context, the regulation of PRB appears to be more sensitive than PRA, considering the fall in E2. It has already been proposed that uterine ER-β, but not ER-α, is detected under low amounts at the cellular level [53]. On the other hand, our data pointed to an increase in ER-α/ER-β ratio. These effects may be due to differential ER expression associated with variations into estrus period. Uterine MT1R was up-regulated after melatonin treatment during ovulation, thereby supporting a direct regulation by melatonin itself. However, it cannot be assumed that melatonin-bound uterine MT1R is involved in down-regulation of ER-β. In contrast, MT1R was found to be depleted after E2 has raised [54], thus demonstrating a negative correlation. Finally, the uterine AR levels seem to be not affected by melatonin during ovulation.

## Conclusions

In summary, we reported that long-term melatonin is able to partially suppress the neuroendocrine reproductive axis during ovulation, indirectly causing disturbances to ovary, oviduct and uterus.

Moreover, melatonin promoted differential regulation of the sex steroid receptors on the reproductive tissues, mostly acting "in situ" through its MT1R receptor (especially in ovarian and uterine tissue) or by altering the dynamics and responsiveness of sex steroid receptor isoforms after binding to E2 or P4. These data represent therefore an important benchmark for furthering the understanding of melatonin-reproduction interface during ovulation process.

## Competing of interests

The authors declare that they have no competing interest.

## Authors' contributions

LGAC, FEM: collected and analyzed the data and drafted the manuscript, beyond conceiving the main idea of the study. FRFS, WJF, GRT, FKD, and AAHF: performed the ELISA assays and Western Blotting analysis given substantial interpretation of data. JPAA, LOM, BAF, MM and PFFP: participated in the acquisition of data, in the design of the study and in the intellectual conception. JAAF: participated in all RIA dosages and during interpretation of these data. The authors helped to perform the statistical analysis. All authors read and approved the final version of the manuscript.
